# Levagen+ (palmitoylethanolamide) alleviates joint pain and reduces the impact of joint pain in canines and felines: a double-blind, placebo-controlled, randomized clinical trial

**DOI:** 10.3389/fvets.2026.1703143

**Published:** 2026-02-13

**Authors:** David Briskey, Ella Craddock, Amanda Rao, Paul C. Mills

**Affiliations:** 1 School of Human Movement and Nutrition Sciences, The University of Queensland, Brisbane, QLD, Australia; 2 RDC Clinical, Brisbane, QLD, Australia; 3 School of Veterinary Science, The University of Queensland, Gatton Campus, Brisbane, QLD, Australia

**Keywords:** canines, felines, joint pain, Levagen+, palmitoylethanolamide (PEA)

## Abstract

**Introduction:**

This study assessed the effectiveness of Levagen+ (palmitoylethanolamide), a fatty acid amide and lipid mediator, for both the alleviation and impact of joint pain, in canines and felines.

**Methods:**

This prospective double-blinded, randomized placebo-controlled study supplemented 50 canines and 50 felines experiencing joint pain daily for 6 weeks with either Levagen+ or a placebo taken orally. Efficacy was determined in canines using the Canine Brief Pain Index (CBPI) and in felines using the Feline Musculoskeletal Pain Index (FMPI), both completed by owners at baseline, week 2, week 4 and week 6. Data were analyzed with distribution-appropriate tests and a two-way repeated-measures ANOVA to assess group, time, and interaction effects across outcomes.

**Results:**

In canines, significantly more were classified as successfully treated in the Levagen+ group compared to the placebo group (76% vs. 40%; *p* < 0.05), with significant improvements in multiple pain and functional interference domains. In felines, significant between-group differences were observed for specific functional tasks [jumping up (*p* < 0.05), jumping down (*p* < 0.05)] and in scores for current pain [week 2 (*p* < 0.05) and week 6 (*p* < 0.05)]. Levagen+ was well tolerated in both species.

**Discussion:**

These findings supported the hypothesis that Levagen+ reduces the impact of joint pain in companion animals.

## Introduction

Pain and chronic pain of joints can have varying aetiologies. One such cause is arthritis, a condition which broadly classifies a range of diseases that occur within bone joints. Clinical signs of arthritis include painful swelling, stiffness and attenuation in the extent of joint motion. Arthritis can be either inflammatory, such as rheumatoid arthritis and lupus, or non-inflammatory, such as osteoarthritis (OA), which is the most common form of arthritis in humans. Osteoarthritis is a chronic synovial joint disease characterized by the gradual degradation and wearing down of articular cartilage over time (1). As cartilage deteriorates, synovial fluid accumulates, and bony outgrowths can form around the joint (1). A 2021 study suggested the world-wide prevalence of OA in humans to exceed 18% (2).

Clinical signs of OA experienced by humans are also evident in canines, with the disease estimated to affect 80% of canines aged over 8 years (3). Current management options include, but are not limited to, nonsteroidal anti-inflammatory drugs (NSAID; e.g., meloxicam), corticosteroids, other classes of analgesics, non-pharmacological therapies (hydro/physio/acupuncture), and stem cell therapy, typically administered either orally or by injection. However, pharmacological management of OA is limited by potential gastrointestinal, renal and hepatic adverse effects, particularly with long-term use or in animals with comorbid disease (4–6). Corticosteroids are typically used only for immediate short-term relief for acute pain, as long-term use may result in adverse effects. Long-term use can cause gastrointestinal side effects such as nausea, vomiting, loss of appetite and diarrhea (7).

Palmitoylethanolamide (PEA) is a fatty acid amide and lipid mediator belonging to the N-acyl-ethanolamine class. PEA is naturally occurring in the body and is found in foods, such as egg-yolk, peanuts and soybeans (7, 8). It can also be formulated and used as a supplement. Studies have shown PEA to have analgesic and anti-inflammatory properties (7, 8). During early research of PEA, the compound was not widely used as a supplement, due to its poor absorption. However, this has been improved by the introduction of absorption enhancing technologies, like LipiSperse™, as used in the current study (7, 8).

PEA is thought to act through multiple pathways, including downregulation of mast cell activation and indirect activation of cannabinoid receptors via the “entourage effect.” It also interacts with peroxisome proliferator-activated receptor-*α* (PPAR-α) to inhibit pro-inflammatory mediator release and restore homeostatic balance in overactive immune cells. However, the scarcity of direct research on PEA’s effects in different species, specifically on canine and feline joint pain, highlights the importance of the current study and the need for further research across a variety of aetiologies and species. By providing evidence of PEA’s efficacy in reducing pain severity and improving functional outcomes in canines and felines with joint pain, this research supports the potential for PEA supplementation as a therapeutic option in canine and feline joint health. PEA has been commonly used in the veterinary setting for the management of dermatitis of canines and felines, but its potential as a supplement to combat joint pain has not yet been widely researched (9).

A previous study investigated the effect of PEA on rats, to determine whether it could be used for the management for joint pain in canines and felines (10). The results suggested that a PEA-quercetin formulation could benefit locomotor function, as well as delay pain onset for rats. A 4-week study tested a PEA-quercetin formulation (24 mg/kg dosage) on canines with identified chronic OA. After 2 weeks, management with PEA reduced the clinical signs of OA in 54.5% of participating canines (11). PEA also successfully reduced Canine Brief Pain Inventory (CBPI) scores, and lameness during the study period. An 8-week study allocated PEA dosages based on each canine’s bodyweight (BW), ranging from 50 mg to 450 mg. PEA supplementation was shown to significantly decreased pruritus visual analog scale scores and increased quality of life scores in canines (12).

A study by Noli et al. (13) found ultra-micronized PEA (PEA-um; 15 mg/kg/day) given to felines for 8-weeks, followed by a 4-week washout, extended relapse time (40.5 vs. 22.2 days) and reduced pruritus severity compared with a placebo. Another study investigated the effects of oral PEA at a dose of 10 mg/kg on 15 felines with eosinophilic plaques and eosinophilic granuloma for 1 month (14). At the end of the study, 67% of felines showed improvements in pruritus, erythema, alopecia, and eosinophilic lesions (14).

The current study aimed to investigate Levagen+ for the management of canines and felines experiencing joint pain. The anti-inflammatory and analgesic actions of PEA in both humans and animals, make it a promising non-pharmaceutical option for improving pain, mobility, and overall quality of life in companion animals. Despite the potential of PEA, robust clinical evidence in canines and felines remains limited, highlighting the need for further investigation. It was hypothesized that Levagen+ would reduce the reported severity and impact of joint pain in companion animals. Effectiveness in canines and felines was measured using the CBPI and Feline Musculoskeletal Pain Index (FMPI) respectively, as a primary outcome.

## Methods

### Design

A randomized, double-blinded, parallel group clinical trial investigating the effectiveness of a Levagen+ supplement on canines and felines over a 6-week period. Canines and felines were recruited though various media, including social media, from across Australia (excluding NSW). Eligible canines and felines were randomized separately using randomization software.^1^ To minimize BW variability affecting the study, canines and felines were separately matched in pairs for BW and randomized into on a 1:1 ratio (Table 1).

**Table 1:** Dose per BW classification for canines and felines.

Species	BW range	Capsules per day (*n*)	Total PEA or placebo per day (mg)
Canine	3.00–6.99 kg	1	150
	7.00–12.99 kg	2	300
	13.00–19.99 kg	3	450
	20.00–34.99 kg	4	600
	>35.00 kg	5	750
Feline	>2.0 kg	1	150

BW, body weight; PEA, palmitoylethanolamide.

Both the animal owner and anyone involved in the study were blinded to the allocation. The study was home-based and took place between July and October of 2024. It complied with the current International Conference on Harmonization (ICH) Guideline for Good Clinical Practice (GCP), the Therapeutic Goods Administration (TGA) Note for Guidance on Good Clinical Practice, as well as the Animal Care and Protection Act 2001 and Animal Care and Protection Regulation 2012. Canine and feline owners provided informed consent prior to commencing the trial. Ethics approval was obtained prior to the start of the study from The University of Queensland Animal Ethics committee (2023/AE000663).

### Study population

50 generally healthy canines and 50 generally healthy felines experiencing joint pain were recruited from across Australia (excluding NSW). Potential canines and felines were screened via their owners against the eligibility criteria. Inclusion criteria included: experiencing joint pain for at least the past 2 months (not related to acute injury), otherwise healthy, minimum BW of 3.0 kg for canine and 2.0 kg for felines, able to take medication when added to food. Exclusion criteria included: unstable or serious illness (a serious illness is a condition that carries a risk of mortality, negatively impacts quality of life and daily function and/or is burdensome in symptoms and/or treatments), known joint deformity (e.g., hip dysplasia), current use of vet-prescribed pain medication, known pregnant or nursing canines or felines, or any condition deemed by the investigator as ineligible. There was no age restriction for canines or felines to participate in this study.

### Trial product

The active product was Levagen+, a commercially available palmitoylethanolamide (PEA) formulation manufactured using LipiSperse^®^ technology to enhance dispersibility and absorption. The placebo consisted of microcrystalline cellulose. Both the Levagen+ and placebo preparations were produced by the same manufacturer (Gencor Pacific Ltd., Hong Kong) to ensure identical taste, appearance, weight, and texture, with matching opaque capsule shells and identical external packaging to maintain blinding for investigators, owners, and assessors. Each capsule contained 150 mg of either Levagen+ or microcrystalline cellulose and dose according to Table 1. The dose selected for this study was based on prior clinical experience with Levagen+ and on dosing regimens successfully used in similar companion-animal trials. For administration, the capsules were opened immediately prior to use, and the contents mixed thoroughly with a portion of the animal’s regular food to encourage complete consumption. Owners were instructed to observe the animal during feeding to ensure the entire portion containing the study product was ingested. Both the Levagen+ and placebo product had minimal taste, therefore ensuring palatability was equivalent and had minimal impact on the canine or felines desire to consume along with its food.

### Procedure

Following screening, canine and feline owners provided written informed consent to enroll their canine or feline into the study. Canines and feline were then randomly allocated to either the Levagen+ or placebo group. Change in joint pain severity was assed throughout the study using validated questionnaire – the CBPI (canines) (15, 16) or FMPI (felines) (17, 18). The CBPI scores 11 domains on a scale of 0 to 10. The FMPI scores 17 domains on a scale of normal to not at all and 2 domains on a sliding scale of no pain to severe pain. Owners completed the CBPI or FMPI at baseline and weeks 2, 4 and 6 to assess the severity of pain and the extent to which the pain interfered with daily function, as observed by the owner. Scoring of the CBPI and FMPI were conducted as per the instructions. Supplementation was classified as successful in the CBPI if the average score of the four pain parameters was ≤ − 1.0 and the average of the average of the six functional interference parameters was ≤ − 2.0. The total FMPI score is the sum of the scores from all the questions, with higher scores indicating greater pain and impairment. Throughout the study period, all owners were asked to monitor and report any adverse events.

### Sample size

Sample size was calculated using G*power (V3.1). Based on matched pair analysis, a minimum of 21 canines and 20 felines were required per group for power to detect a 20–25% reduction in CBPI pain scores (i.e., an average of 6 ± 2 at baseline reduced to 4.5 ± 2 at end of study, effect size 0.75, power 95%, alpha error 0.05) or FMPI (i.e., an average of 36 ± 10 at baseline reduced to 29 ± 8 at end of study, effect size 0.76, power 95%, alpha error 0.05). Therefore 25 canines and 25 felines were recruited to each group (50 total) to allow for a 20% dropout rate.

### Statistical analysis

Conducted using GraphPad Prism 8.0 and SPSS with only complete data sets included. All data was first assessed for distribution to allow for the correct test selection. Normally distributed variables were analyzed using independent-samples *t*-tests, while non-normally distributed variables were analyzed using Mann–Whitney *U* tests. A two-way repeated-measures ANOVA (factors: group, time, and group × time interaction) was also performed to evaluate overall treatment effects across the four assessment time points. Each individual CBPI and FMPI domain, as well as the average pain score, CBPI total score, and total FMPI score, were analyzed for between-group differences at each time point. Significance was set at *p* < 0.05, with *p* ≥ 0.05 considered non-significant.

## Results

**Figure 1:**
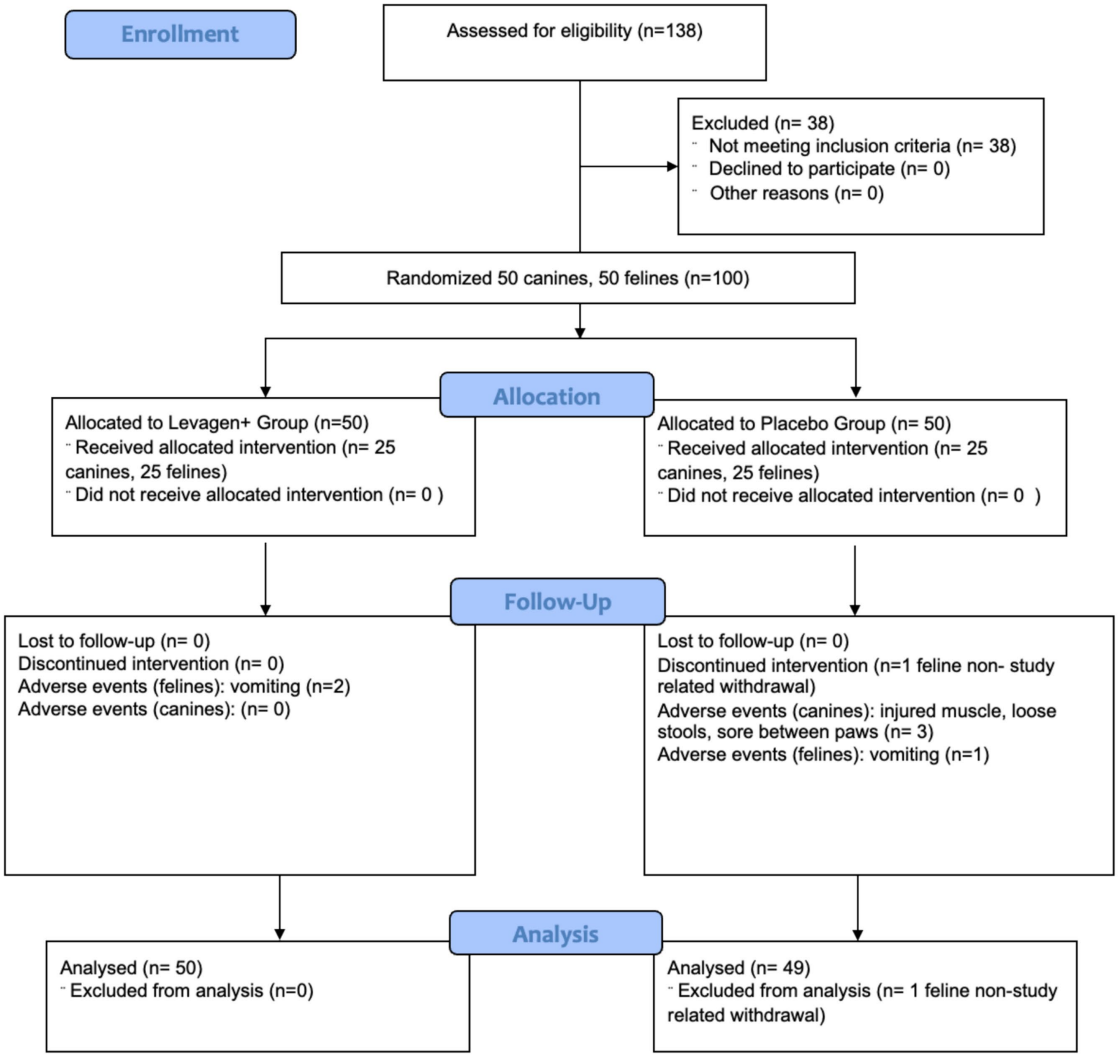
CONSORT flow diagram illustrating participant recruitment, allocation, follow-up, and analysis for the trial.

50 canines and 50 felines were enrolled in the trial and all 50 canines and 49 felines completed all study requirements. In the canines, three adverse events were reported in the placebo group (injured muscle, loose stools and developed a sore between the paws) and no adverse events were reported in the Levagen+ group. In the felines, three adverse events of vomiting were reported (2 Levagen+ and 1 placebo). Of the 50 canines and 50 felines in the study, one feline in the placebo group withdrew (not study related) and was not included in the analysis (Figure 1). At baseline, there was no difference between the respected groups for age, BW or BW group distribution for either canines or felines (Table 2).

**Table 2:** Demographics and BW distribution for canines and felines.

Specie	Demographic	Levagen+ (*n* = 25)	Placebo (*n* = 25)
Canines	Age (years; mean ± SD)	9.8 (3.2)	10 (2.5)
	Average BW (kg)	21.8 (13.4)	22.2 (15.9)
	3.00–6.99 kg (n)	6	6
	7.00–12.99 kg (n)	3	3
	13.00–19.99 kg (n)	4	4
	20.00–34.99 kg (n)	6	6
	>35 kg (n)	6	6
Felines	Age (years; mean ± SD)	10.2 (4.2)	11.3 (4.9)
	Average BW (kg)	4.9 (2.1)	4.7 (1.5)

SD, standard deviation; BW, body weight.

### Canine results

At baseline, there was no significant difference between groups for in the CBPI scores (Table 3). Following supplementation, significantly more canines were classified as successfully treated in the Levagen+ group compared to the placebo group (Table 3; *p* < 0.05). Specifically, the Levagen+ group showed a significant reduction in CBPI pain scores compared to the placebo for: pain at its worst (weeks 4 and 6), pain at its least (week 6), average observed pain (week 6), pain right now (week 6) and the average pain scores (week 6). Levagen+ also showed a significant reduction in CBPI pain functional interference scores compared to the placebo for: general activity (week 4 and 6), Enjoyment of life (week 4), ability to rise to standing from laying down (week 4 and 6), ability to walk and run (week 2, 4 and 6) and pain interference score (week 4 and 6). No significant difference was seen throughout the study for overall quality of life.

**Table 3:** Description of pain as measured by Canine Brief Pain Inventory (CBPI).

CBPI measure	Levagen+ (*n* = 25)				Placebo (*n* = 25)				*p*-value^a^
	Baseline	Week 2	Week 4	Week 6	Baseline	Week 2	Week 4	Week 6	
Pain (last 7 days)									
Pain at its worst	5.7 (1.3)	3.9 (1.8)	3.2 (1.5)^#^	2.7 (2.1)*	5.8 (1.8)	4.7 (2.0)	4.5 (2.3)	4.1 (2.5)	0.022
Pain at its least	3.2 (2.1)	2.2 (1.7)	1.5 (1.6)	1.2 (1.0)*	3.2 (1.7)	2.4 (1.9)	2.1 (2.0)	2.0 (1.8)	0.031
Average pain	4.6 (1.5)	3.0 (1.5)	2.4 (1.5)	1.8 (1.4)^#^	4.6 (1.4)	3.5 (1.7)	3.1 (1.8)	3.1 (2.0)	0.007
Pain right now	3.8 (2.0)	2.4 (1.8)	2.2 (1.6)	1.3 (1.4)*	4.1 (2.0)	2.5 (2.1)	2.4 (2.0)	2.6 (2.6)	0.012
*Average pain score*	4.3 (1.4)	2.9 (1.5)	2.3 (1.4)	1.8 (1.3)*	4.4 (1.5)	3.3 (1.7)	3.0 (1.8)	2.9 (2.1)	0.020
Function interference (last 7 days)									
General activity	5.3 (1.5)	3.1 (1.8)	2.4 (1.5)*	1.8 (1.7)^#^	4.6 (2.2)	3.5 (2.1)	3.4 (2.2)	3.2 (2.2)	0.009
Enjoyment of life	4.2 (1.6)	2.5 (2.2)	1.7 (1.6)*	1.5 (1.6)	4.4 (2.5)	3.0 (2.1)	2.8 (2.1)	2.4 (2.5)	0.072
Ability to rise to standing from lying down	5.5 (2.0)	3.1 (1.9)	2.7 (2.1)*	2.0 (1.8)^#^	4.7 (2.5)	3.3 (2.2)	3.9 (2.1)	3.6 (2.5)	0.009
Ability to walk	4.0 (2.2)	2.3 (1.8)*	2.2 (1.9)*	1.5 (1.8)^#^	4.6 (2.7)	3.7 (2.1)	3.4 (2.4)	3.3 (2.7)	0.003
Ability to run	5.2 (2.4)	3.2 (2.2)*	2.5 (1.9)^#^	2.3 (1.9)*	6.2 (28)	4.6 (2.4)	4.1 (2.5)	3.6 (2.8)	0.025
Ability to climb stairs	5.5 (2.4)	3.7 (2.4)	3.2 (2.5)	2.6 (2.4)	5.7 (2.5)	4.5 (2.8)	4.1 (2.5)	3.3 (2.5)	0.162
*Pain interference score*	5.0 (1.6)	3.0 (1.9)	2.5 (1.8)*	1.9 (1.7)*	5.0 (2.1)	3.8 (2.0)	3.6 (2.1)	3.2 (2.3)	0.016
*Successful* [*n* (%)]	–	–	–	19 (76.0)*	–	–	–	10 (40.0)	0.017
Quality of life	3.9 (0.8)	4.2 (0.9)	4.5 (0.7)	4.7 (0.9)	3.7 (0.9)	4.1 (0.9)	4.1 (0.8)	4.5 (1.0)	0.218

a *p*-values presented are between group differences at week 6. Statistically significant compared to placebo * = *p* < 0.05; ^#^ = *p* < 0.01; unless specified, values presented are mean (SD).

**Figure 2:**
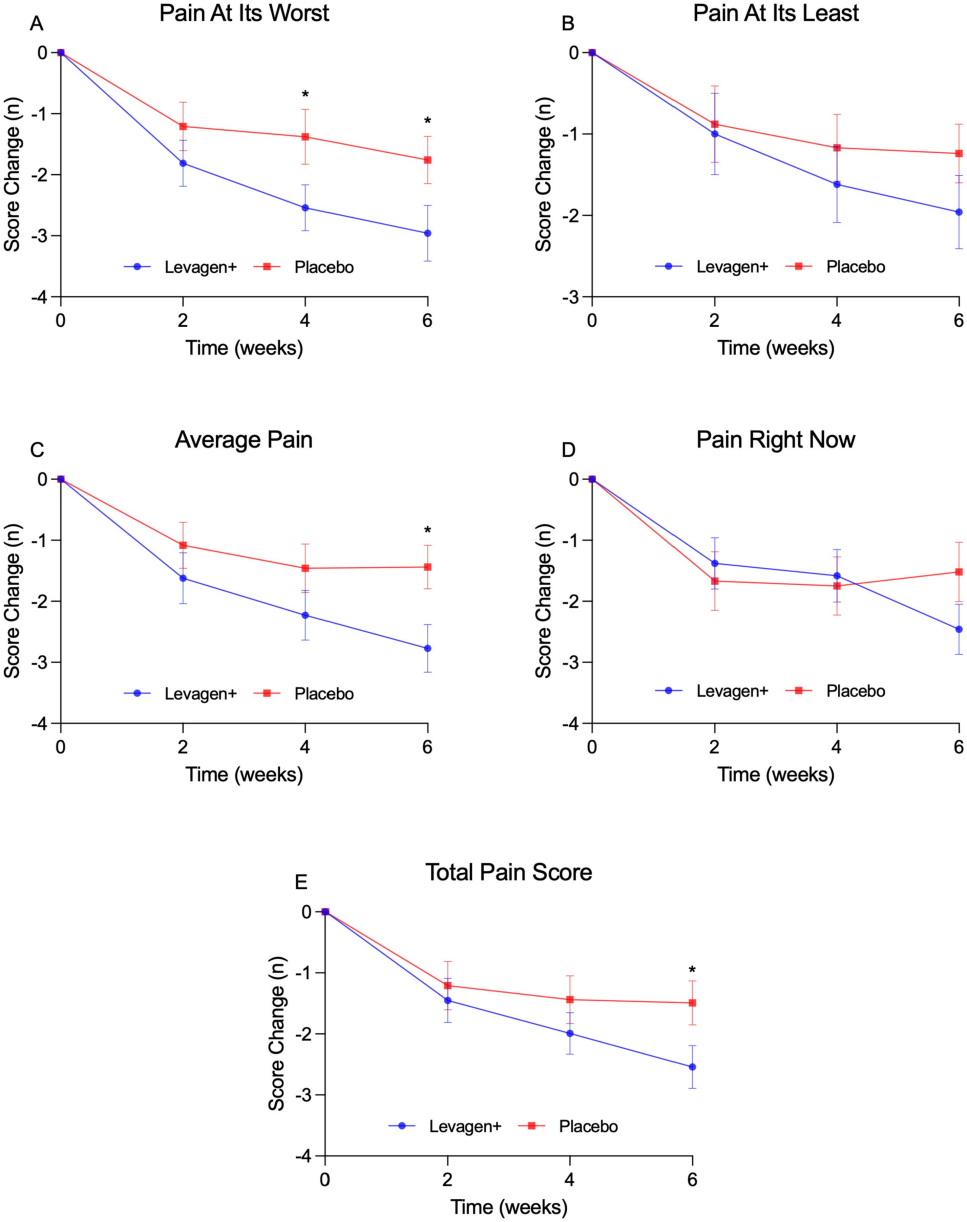
CBPI change in pain scores over 6 weeks. **(A–E)** Pain at its worst **(A)**, pain at its least **(B)**, average pain **(C)**, pain right now **(D)**, total pain scores **(E)**. * = significant difference between groups (*p* < 0.05); values presented are change from baseline values with SEM.

**Figure 3:**
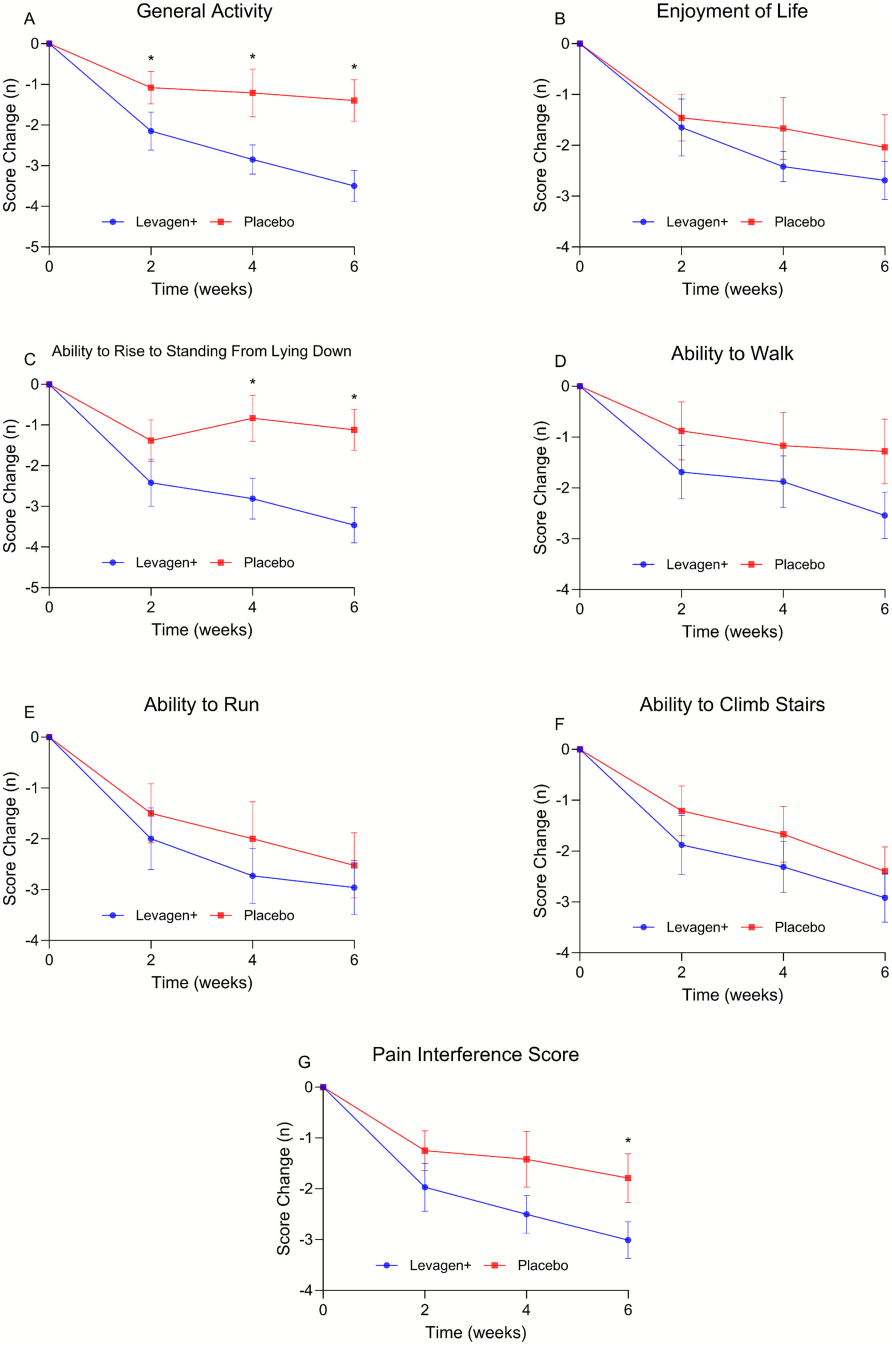
Change in pain interference scores from CBPI. **(A)** General activity, **(B)** enjoyment of life, **(C)** ability to rise to standing from lying down, **(D)** ability to walk, **(E)** ability to run, **(F)** ability to climb stairs, **(G)** pain interference score. * = significant difference between groups (*p* < 0.05); values presented are change from baseline values with SEM.

When data was adjusted for change from baseline, Levagen+ supplementation significantly reduced the observed pain at its worst at week 4 (−2.5 ± 1.9 vs. −1.4 ± 2.1) and week 6 (−3.0 ± 2.3 vs. −1.8 ± 1.9), average observed pain at week 6 (−2.8 ± 2.0 vs. −1.4 ± 1.7) and average of pain scores at week 6 (−2.5 ± 1.8 vs. −1.5 ± 1.7) compared to the placebo group (Figures 2A–E). Levagen+ significantly reduced the impact of pain on general activity at week 2 (−2.2 ± 2.4 vs. −1.1 ± 1.8), week 4 (−2.9 ± 1.8 vs. −1.2 ± 2.7) and week 6 (−3.5 ± 1.9 vs. −1.4 ± 2.5), ability to rise to standing from lying down at week 4 (−2.8 ± 2.5 vs. −0.8 ± 2.7) and week 6 (−3.5 ± 2.2 vs. −1.1 ± 2.5), and pain interference score at week 6 (−3.0 ± 1.8 vs. −1.8 ± 2.3) (Figures 3A–G).

### Feline results

**Figure 4:**
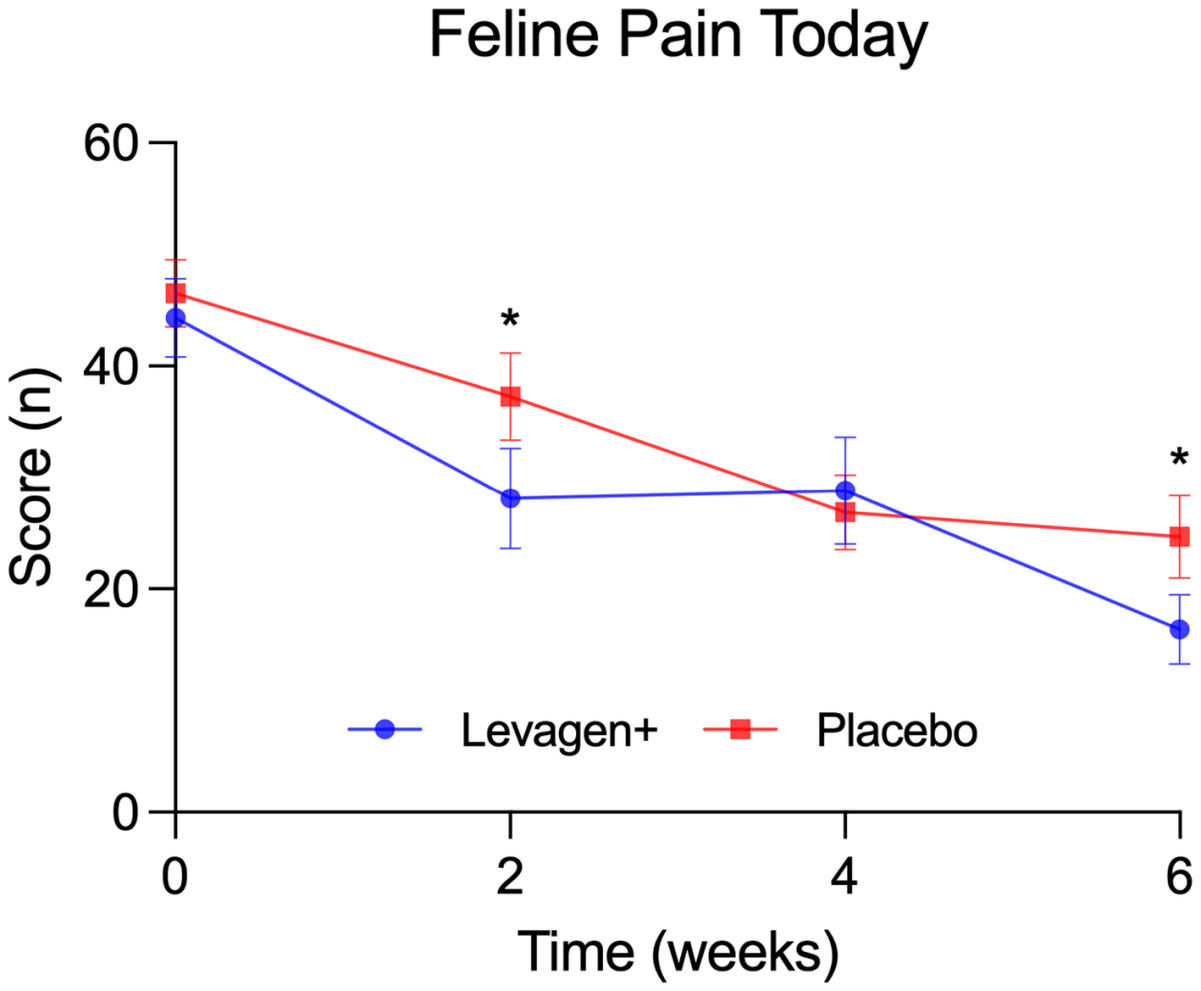
Feline pain score reported for that day. * = Significant difference between groups (*p* < 0.05); values presented are absolute values with SEM.

From the FMPI data, scores for current pain (pain today) significant improvements in the Levagen+ group compared to the placebo group at both week 2 and week 6 (Figure 4). While overall FMPI scores improved in both groups, the difference between groups did not reach statistical significance. Item-by-item analysis of the FMPI revealed notable benefits in the Levagen+ group, with statistically significant improvements by week 6 in two key functional measures — the ability to jump up and the ability to jump down (Table 4).

**Table 4:** Feline musculoskeletal pain index scores reported.

FMPI measure	Levagen+ (*n* = 25)				Placebo (*n* = 24)			
	Baseline	Week 2	Week 4	Week 6	Baseline	Week 2	Week 4	Week 6
Walk and move easily	1.24	0.72	0.88	0.52	1.00	0.63	0.58	0.67
Run	1.56	1.08	1.12	0.76	1.83	1.30	1.04	1.13
Jump up	1.84	1.48	1.24	0.72*	1.88	1.50	1.38	1.17
Jump up to kitchen counter	2.57	2.29	1.71	1.41	3.13	2.22	1.82	1.91
Jump down	1.74	1.41	0.96	0.80*	2.25	1.42	1.38	1.30
Climb up stairs or steps	1.44	0.82	0.67	0.48	1.61	0.88	0.67	0.59
Go down the stairs or steps	1.25	0.82	0.61	0.57	1.44	0.88	0.61	0.47
Play and/or chase objects	1.71	1.12	1.46	1.00	2.09	1.52	1.05	1.05
Play with other pets	1.74	0.78	1.00	0.60	2.29	1.21	0.89	1.16
Get up from resting	1.32	0.52	0.68	0.52	1.35	0.88	0.54	0.54
Lie and or sit	0.72	0.24	0.48	0.44	0.75	0.42	0.21	0.42
Stretch	0.76	0.25	0.67	0.40	1.13	0.67	0.50	0.67
Groom themselves	0.80	0.64	0.76	0.44	0.96	0.75	0.67	0.58
Interact with you	0.40	0.40	0.48	0.16	0.63	0.29	0.21	0.29
Tolerate being touched	0.80	0.60	0.40	0.40	1.17	0.42	0.79	0.54
Eat	0.28	0.24	0.16	0.16	0.08	0.08	0.17	0.21
Use the litter box	0.72	0.41	0.17*	0.17	0.68	0.46	0.52	0.32
Total FMPI	18.9	12.4	12.5	9.1	22.9	14.5	12.1	12.0
Pain over last 2 weeks	44.3	33.1	32.6	20.1	49.2	40.8	30.3	26.3

* Statistically significant compared to placebo; *p* < 0.05. FMPI, Feline Musculoskeletal Pain Index.

## Discussion

The findings from this study showed that supplementation with Levagen+ is safe, well tolerated, and can significantly improve pain and function in canines and felines with joint pain. Over the 6-week study period, the canine Levagen+ group showed a consistent reduction in pain and functional interference scores, indicating an efficacy for Levagen+ supplementation to alleviate joint pain and its impact on function. Notably, pain at its worst decreased significantly from week 4, suggesting an early and sustained analgesic effect. While in felines, the Levagen+ group showed significant improvement in specific functional tasks and pain levels at week 6, compared to the placebo group.

In addition to pain reduction, Levagen+ significantly improved functional outcomes related to pain interference in both canine and felines. In canines, improvements were observed as early as week 2 and maintained through weeks 4 and 6. The findings highlight a potential rapid onset of functional improvement with Levagen+ supplementation. While in felines, improvements were seen at week 6 and indicated improvements in critical functional measures of jumping up and down. These outcomes indicated improvements in critical indicators of mobility in both canines and felines with joint pain, highlighting the functional benefits achieved by Levagen+ supplementation.

To date, evidence to support the efficacy of PEA for canine and feline joint pain and function remains limited. The objective of this study was to add to the growing body of research supporting the anti-inflammatory and analgesic properties of Levagen+/PEA across a broader range of conditions and species. Our findings align with the only known comparable canine study in which dogs were supplemented with PEA-um with quercetin (24 mg/kg BW). In that trial, thirteen medium-to-large breed dogs with chronic OA and persistent lameness received supplementation for 4 weeks. Assessment using the CBPI revealed management of symptoms was classified as successful in 54.5% of canines as early as week 2, with CBPI scores continuing to show significant improvement throughout the study period. Lameness, whether scored by a veterinarian or assessed objectively, also showed significant improvement (19).

The results from this study were also comparable to studies that have utilized pharmaceutical interventions in canines with joint pain. In a study by Brown et al. (16), the CBPI was used in a double-blind, randomized, placebo-controlled trial involving 70 canines with OA. Canines treated with carprofen, a NSAID, showed significant improvements in median pain severity scores, decreasing from 4.25 at baseline to 2.25 (−2.0 change) after 14 days. Pain interference scores also improved from 4.33 to 2.67 (−1.76 change) during the same period. In contrast, the placebo group showed no significant changes, with pain severity scores of 3.50 at baseline and 3.25 (−0.25 change) at day 14, and pain interference scores of 3.92 at baseline and 3.25 (−0.67 change) at day 14.

A study by Salichs et al. (20) compared NSAIDs to a placebo for the management of canine OA over a 42-day period. While specific CBPI values were not stated, the study reported that 90% of canines treated with enflicoxib (*n* = 78) and 79% treated with mavacoxib (*n* = 80) were classified as CBPI responders, compared to 43% in the placebo group (*n* = 22). This indicated a significant improvement in pain and function among canines receiving the active products.

Comparatively, the current study showed similar outcomes with the Levagen+ group having a 76% success compared to the placebo’s 40%. The magnitude of pain reduction observed with Levagen+ supplementation is equivalent to, and in some aspects, superior to outcomes reported in similarly studies utilizing the CBPI to evaluate pain management and/or involving NSAIDs and other analgesic agents in canines with joint pain.

To our knowledge, this is the first study to investigate the efficacy of PEA on joint pain in felines. When compared to other studies of similar design, our results showed some similarities to a clinical trial evaluating oral glucosamine/chondroitin sulfate supplementation in cats with joint pain (21). Both the Levagen+ and placebo groups showed within-group improvements over 8 weeks in FMPI and client-specific outcome measures, but statistically significant between-group differences were not found (21). These findings underscored the potential influence of placebo responses in owner-reported outcome measures and the difficulty of detecting supplement effects when using composite scores that may be less sensitive than specific functional endpoints. From a clinical perspective, improvements in jumping ability are particularly noteworthy. The ability to restore such movements suggests that PEA may positively influence both pain and functional capacity, translating into a better quality of life for affected cats.

The observation in felines that significant benefits were detected in jumping ability, but not in the composite FMPI score, highlighted the importance of examining specific functional items rather than relying solely on total scores. This is supported by previous work demonstrating that certain FMPI questions, such as those relating to jumping up and down, are more responsive to change than others and are strongly correlated with objective measures of mobility (22).

Our findings were also supported by other studies incorporating PEA and animal models of joint pain. An animal study investigated a combination of PEA and quercetin (20 mg/kg) in rats demonstrated an efficacy in decreasing inflammation and relieving pain in inflammatory and OA pain models (10). This study involved administering PEA and quercetin three times per week for 18 days after sodium monoiodoacetate-induced OA. This study showed that PEA can have efficacy even when not taken daily and in lower quantities than that dosed in the current study. And while the results supported our data, it is difficult to directly compare the study outcomes due to the variability in conditions.

One limitation of the current study was the ability to accurately measure a canine’s or felines pain level, and their impairment from pain. The subjective nature of the CBPI and FMPI questionnaires make them both reliant upon the owner’s accurate observation of their canine’s perceived pain. This may be further influenced by factors, such as the amount of time the owner spends observing their canine or feline. The absence of statistically significant between-group differences in aspects of both the CBPI and FMPI scores may also reflect limitations in sample size, study duration, or the sensitivity of the instrument to detect subtle effects. Future research might consider incorporating both subjective and objective outcome measures, such as activity monitors, to complement owner-reported data. Longer follow-up periods may also help determine whether the observed improvements translate into sustained mobility benefits, and if the placebo effect dissipates over time. To better support the clinical suitability of PEA, future studies incorporating the comparison with current standard of care paired with veterinary assessments would be of importance.

These findings of the current study align with patterns observed in other canine and feline joint pain intervention studies. Overall, this study provided evidence that Levagen+ supplementation is effective in reducing pain severity and improving functional outcomes in canines and felines with joint pain. These findings suggested that PEA supplementation offers a promising alternative to traditional medications, with comparable efficacy in alleviating joint pain and improving functional outcomes in canines observed as early as week 2 and in felines at week 6. These findings supported the potential for Levagen+ as a therapeutic option for enhancing the daily function of canines and felines experiencing joint-related pain and functional limitations.

## Data Availability

The raw data supporting the conclusions of this article will be made available by the authors, without undue reservation.

## Footnotes

1
www.sealedenvelope.com
